# Assessing adherence and identifying barriers to breast cancer screening among females in the central region (Riyadh) of Saudi Arabia: a cross-sectional study

**DOI:** 10.3389/fpubh.2026.1924881

**Published:** 2026-10-15

**Authors:** Jehad A. Aldali

**Affiliations:** Department of Pathology, College of Medicine, Imam Mohammad Ibn Saud Islamic University (IMSIU), Riyadh, Saudi Arabia

**Keywords:** barriers, breast cancer, knowledge, mammography, screening adherence

## Abstract

**Background:**

Breast cancer is a frequent female malignancy worldwide. Central Saudi women can benefit from breast cancer screening. This study examines adult women’s adherence to breast cancer screening standards and the main impediments to participation.

**Methods:**

A cross-sectional online survey was conducted in the Central Region (Riyadh) of Saudi Arabia from 13 September 2025 to 28 February 2026.

**Results:**

404 people took the survey. The majority of participants were younger than 30 years (37.9%), followed by participants aged 40–49 (23.8%). Most participants (96.5–99.5%) endorsed the listed benefits of breast cancer screening, whereas 66.1% reported knowing how to screen for breast cancer. Most participants supported screening even in the absence of symptoms (86.4%) and reported that they would follow a doctor’s recommendation (84.9%). Regular mammography was reported by 21.5% of the full sample and by 43.1% of participants aged ≥40 years. The most frequently reported barrier was believing that screening was unnecessary in the absence of symptoms (69.8%), followed by fear of examination (55.2%), time constraints (49.0%), and fear of the results (48.8%).

**Conclusion:**

Participants generally recognized the importance of breast cancer screening, but screening practices remained limited. Commonly reported concerns included the belief that screening was unnecessary without symptoms, fear of examination and results, and time constraints. These findings suggest that awareness alone may not be sufficient to improve screening uptake.

## 1 Introduction

Breast cancer continues to be a significant public health concern both in Saudi Arabia & globally, with its early identification significantly reliant on adequate screening techniques, notably mammography (1). Breast cancer cells develop when abnormal changes cause breast cells to grow and divide uncontrollably, potentially invading surrounding tissues or spreading to other parts of the body. Understanding the common signs and risk factors of breast cancer, together with awareness of evaluation methods such as breast self-awareness, clinical breast examination, mammography, ultrasound, and biopsy, can help women recognize suspicious changes and seek appropriate medical assessment at an early stage (2, 3). Breast cancer is a predominant cause of mortality in females, and early identification is associated with reduced morbidity and mortality related to this condition (4). The highest incidence occurs during the menopausal transition, a period often marked by hormonal changes. Accordingly, Saudi national guidelines recommend a regular mamm breast cancer screening ography screening every 1–2 years for women aged 40–69 years (5, 6). Even though the age recommended for screening starts from 40, the Breast Cancer Screening Program, permits women from aged 20 and above.

Health care workers in Saudi Arabia should stress how important it is to be aware of and recognize changes in the breasts (7, 8). According to the current American Cancer Society (ACS) guidelines, routine clinical breast examination by a healthcare professional is not recommended for breast cancer screening among average-risk women of any age. This differs from the 2003 ACS guidelines, which recommended an examination approximately every 3 years for women aged 20–39 and annually from age 40 and older. The updated 2015 ACS guidelines for breast cancer screening among women at average risk instead emphasize age-based mammography (7, 8). Due to the danger of breast cancer, it is critical to adhere to the breast cancer screening guidelines among adult females in Saudi Arabia and to identify the key barriers that hinder effective participation in these screening programs.

Across Europe, females between the ages of 50 and 69 who do not have any symptoms are invited to receive a routine breast cancer screening every 2 years as part of organized public health programs. A mammogram is the most common test for detecting breast cancer in its early stages, even before any physical signs appear. By capturing low-dose X-ray images of breast tissue, mammography can uncover tiny calcifications or small tumors that cannot be felt during a manual exam. Catching these changes early dramatically increases the chances of successful treatment and allows for less invasive therapeutic options (9, 10). Studies in Spain have demonstrated that this type of screening reduces the death rate from breast cancer by 9 to 15% (11).

In Saudi Arabia, breast cancer is the most prevalent malignancy among females, constituting a substantial proportion of female cancers (12). Saudi Arabia has nationwide breast cancer screening programs October 23, 2007 targeted females aged 40 years and older. The national Saudi breast cancer screening guidelines provide an important framework for understanding the recommended age groups, screening practices, and the role of early detection in reducing the disease burden (13), although adherence to screening is low and data remain scarce. Lack of comprehensive, population-based data shows a gap in understanding real-world adherence and the main barriers to breast cancer screening in Saudi Arabia’s adult women.

In Saudi Arabia central region breast cancer screening cases are shaped by geography, because of the access to breast cancer screening services, diagnostic follow-up, health infrastructure, and public awareness often vary across cities, urban and rural settings, and different population groups (14). These geographic differences can influence when cancer is detected, participation in screening programs, and overall outcomes (15). Establishing the geographical context is therefore important to the study, as it helps explain local patterns of screening uptake, identify potential disparities in access and care, and support interpretation of the findings within the specific setting under examination (14, 15).

Although information and positive attitudes toward breast cancer screening are important, they may not be sufficient to ensure actual adherence, as women’s screening behavior is influenced by psychological, social, cultural, and practical barriers (16). This study is conceptually guided by the Health Belief Model and the Theory of Planned Behavior, which help explain why awareness may not always translate into action. According to the Health Belief Model, women are more likely to participate in screening when they perceive themselves as susceptible to breast cancer, recognize its seriousness, believe in the benefits of early detection, and perceive barriers as manageable (17). However, fear of diagnosis, embarrassment, misconceptions, low perceived risk, concerns about pain, limited access, or lack of family support may prevent screening even among women who are aware of its importance. Similarly, the Theory of Planned Behavior suggests that screening is shaped not only by attitudes but also by subjective norms and perceived behavioral control, including social approval, cultural expectations, healthcare provider encouragement, appointment availability, transportation, and confidence in navigating health services (18). Therefore, adherence to breast cancer screening among females adults in the Central Region of Saudi Arabia should be understood as the result of interacting individual, social, cultural, and healthcare-system factors, making it essential to identify the barriers that prevent knowledge and favorable attitudes from being implemented into preventive screening behavior (19).

This study aims to evaluate adherence to breast cancer screening guidelines among adult women in the Central Region (Riyadh) of Saudi Arabia and to identify the primary barriers that impede effective participation in these screening programs. For this study, the target population is operationally defined as adult women who are eligible for breast cancer screening, are willing to participate, and have no prior diagnosis of breast cancer. By examining factors such as awareness, cultural attitudes, accessibility of screening services, and socioeconomic status, this study seeks to provide essential evidence to inform public health policies and improve the uptake of breast cancer screening, ultimately supporting early detection and better management of breast cancer in the Saudi population.

## 2 Materials and methods

### 2.1 Study design and participants

A cross-sectional survey was conducted in Riyadh (Central Region), Saudi Arabia, from 13 September 2025 to 28 February 2026. The Females living in central Saudi Arabia can access non-invasive breast cancer screening through public primary healthcare centers (PHCs), where these services are provided free of charge under the national Breast Cancer Screening Program. The study targeted females aged over 20 years, irrespective of nationality, to evaluate adherence to and barriers to Breast Cancer Screening. Participants who had previously been screened for breast cancer were not excluded from the study, unless they had a prior diagnosis of breast cancer, participants who were unwilling to participate were excluded.

### 2.2 Inclusion and exclusion criteria

These eligibility criteria defined the population relevant to the research question. Duplicate responses were identified and removed separately during data cleaning to ensure that each participant was represented only once in the analysis.

#### 2.2.1 Inclusion criteria

- Female participants aged 18 years or older who were residing in the Central Region (Riyadh) of Saudi Arabia at the time of the study were eligible to participate.
- Participants were required to be able to understand the survey questions, provide informed consent, and complete the online questionnaire.
- females were included regardless of whether they had previously undergone breast cancer screening, as the study aimed to assess both screening adherence and perceived barriers to screening.

#### 2.2.2 Exclusion criteria

- Participants were excluded if they were younger than 18 years, were not female, or were not residents of the Central Region (Riyadh) of Saudi Arabia.
- Females who did not provide informed consent or submitted substantially incomplete questionnaires were also excluded.
- Duplicate responses, when identified, were removed to ensure that each participant was represented only once in the final analysis.

### 2.3 Ethical considerations

The study protocol was approved by the Institutional Review Board (IRB) of Imam Mohammad Ibn Saud Islamic University (project number 879–2025; approval date: 11 September 2025). Participation was voluntary, and electronic informed consent was obtained from each participant before inclusion. All participant data was treated confidentially and securely, with access restricted to the research team.

### 2.4 Data collection and sample size

A cross-sectional study was conducted using a self-administered electronic questionnaire created in Google Forms. Portions of the questionnaire were adapted from prior research (20). Before the main data collection, the survey was pilot tested on 10 faculty members to assess clarity, content validity, and any technical issues. The questionnaire was designed to be concise, and participants were informed that the information provided would be used only for research purposes (suitable for the study objectives), among females adults in the Central Region of Saudi Arabia. The survey was distributed electronically in the Central Region of Saudi Arabia.

The survey was distributed electronically in the Central Region of Saudi Arabia. Participants were recruited through several channels to improve the reach and diversity of responses. These included institutional mailing lists (31.4%), social media platforms such as Twitter and the female faculty WhatsApp (45.1%), and direct peer-to-peer snowball sharing (23.5%). The survey was also advertised via a brief invitation email sent by the university health centre to female faculty staff. Describing the study objective, eligibility criteria, and the survey link. Although online surveys may overrepresent individuals who are more digitally literate or health-conscious, the use of multiple recruitment strategies helped broaden participant reach. Google Forms settings were also used to limit duplicate responses.

The required sample size was calculated using the Raosoft sample size calculator to be at least 385 participants, based on a 95% confidence level and a 5% margin of error, to ensure adequate power for statistical analysis.

A questionnaire adapted from prior research was used to assess participants’ knowledge, attitudes, perceived barriers, and screening practices related to breast cancer screening. Knowledge was assessed using 8 items, including one self-rated item measured on a 10-point scale (e.g., “How would you rate your knowledge of breast cancer screening?”) and seven Yes/No items assessing screening benefits and practical knowledge (e.g., “Do you know how to screen for breast cancer?”). Pro-screening attitude was assessed using 4 items, including two Yes/No items (e.g., screening even without symptoms and willingness to follow a doctor’s recommendation), one 10-point item measuring support for screening after age 45, and one negatively worded Yes/No item assessing skepticism about test effectiveness (reverse-coded). Barriers were assessed using one multiple-response question containing nine barrier options (e.g., fear of examination, cost, or believing screening is unnecessary in the absence of symptoms), and participants could select more than one option. Each option was coded as 1 if selected and 0 if not selected, and the nine indicators were summed to form the barriers index. Yes/No items were coded as (0 = No, 1 = Yes). For composite scoring, the two 10-point items were dichotomized using predefined cut-offs (reported in the notes to Tables 1, 2), and the negatively worded skepticism item was reverse-coded so that higher values reflected greater trust in screening effectiveness. Composite indices were computed as summed scores: knowledge (0–8), pro-screening attitude (0–4), barriers (0–9), and screening practices (0–2). In the full analytical sample (*N* = 404), internal consistency was assessed for the composite indices, with Cronbach’s alpha values of 0.741 (knowledge), 0.720 (attitude), 0.793 (barriers), and 0.748 (practices).

**Table 1:** Knowledge about breast cancer screening (*N* = 404).

Item	Response	*N*	%
How would you rate your knowledge of breast cancer screening	Low	228	56.4%
	High	176	43.6%
Whether each of the following was a benefit of breast cancer screening (yes/no):			
[Early detection of breast cancer]	No	3	0.7%
	Yes	401	99.3%
[Improving chances of cure and successful treatment]	No	2	0.5%
	Yes	402	99.5%
[Reducing the risk of death from breast cancer]	No	14	3.5%
	Yes	390	96.5%
[Providing reassurance when results are normal]	No	4	1.0%
	Yes	400	99.0%
[Detecting changes or lumps that may become dangerous later]	No	14	3.5%
	Yes	390	96.5%
[Preventing the development of cancer]	No	6	1.5%
	Yes	398	98.5%
Do you know how to screen for breast cancer?	No	137	33.9%
	Yes	267	66.1%

Values are *n* (%). The item “How would you rate your knowledge of breast cancer screening” was originally measured on a 1–10 scale (M = 5.26, SD = 2.94; range = 1–10) and was recoded into a binary indicator for composite scoring (1 if ≥6, 0 otherwise).

**Table 2:** Attitudes toward breast cancer screening (*N* = 404).

Item	Response	*N*	%
Do you think breast cancer screening should be done even if you do not have symptoms?	No	55	13.6%
	Yes	349	86.4%
How strongly do you support screening after age 45?	Not strong	139	34.4%
	Strong	265	65.6%
If your doctor recommends a test, will you follow it?	No	61	15.1%
	Yes	343	84.9%
Do you question the effectiveness of the test? (Reverse-coded)	No	378	93.6%
	Yes	26	6.4%

Values are *n* (%). The item “How strongly do you support screening after age 45?” was originally measured on a 1–10 scale (M = 7.81, SD = 2.93; range = 1–10) and was recoded into a binary indicator for composite scoring (1 if ≥8, 0 otherwise). The item “Do you question the effectiveness of the test?” is negatively worded and was reverse-coded (i.e., “No” indicates higher trust).

### 2.5 Data analysis

The statistical analysis was performed using SPSS (IBM version 27). Categorical variables were summarized as frequencies and percentages, while continuous variables were summarized as means (M) and standard deviations (SD) with ranges where appropriate. The primary outcome was regular breast cancer screening assessed by the item “Have you had a mammogram regularly?” (0 = No, 1 = Yes). Bivariate associations between regular breast cancer screening and participant characteristics were examined using Pearson’s chi-square test. As a secondary age-restricted analysis, regular mammography uptake was also summarized separately for participants aged ≥40 years and those aged <40 years to contextualize the full-sample uptake estimate given the age distribution of the sample; the two age groups were compared using Pearson’s chi-square test. Spearman’s rank-order correlations were used to examine associations between the barriers score and the knowledge, pro-screening attitude, and screening practices scores. Descriptive subgroup summaries of the knowledge, pro-screening attitude, and barriers scores were generated according to family history of breast cancer, history of breast disease, and discussion of breast cancer with a doctor. Predictors were selected *a priori* based on the study objectives and prior literature and entered simultaneously into the multivariable model (Enter method). The model included age, education, salary income, healthcare payment method, perceived appropriate age to start screening, family history of breast cancer, history of breast disease, discussion of breast cancer with a doctor, knowledge score, pro-screening attitude score, and barriers score. This approach was used to estimate mutually adjusted associations while accounting for potential confounding by the other variables included in the model. A multivariable binary logistic regression model was then used to estimate adjusted odds ratios (aORs) with 95% confidence intervals (CIs).

## 3 Results

In this study, 408 participants were involved, but 4 were eliminated because they did not meet the inclusion criteria (because they were incomplete). Therefore, the final sample size was reduced to 404 participants. The majority of participants were younger than 30 years (37.9%), followed by participants aged 40–49 (23.8%), while those older than 60 represented the smallest group (4.2%). Most participants held a bachelor’s degree (50.2%), followed by high school education (35.6%). More than half of the sample reported earning less than 5,000 SAR (56.9%), while (20.0%) reported 5,000–10,000. For healthcare payment, government sponsorship was the most common (45.0%), followed by private health insurance (28.2%). Finally, discussion of breast cancer with a doctor was relatively uncommon, with 23.3% reporting having discussed it and 76.7% reporting no discussion. This question was asked of all participants, regardless of previous breast disease or prior breast cancer screening. This is summarised in Table 3.

**Table 3:** Demographic characteristics of the participants (*N* = 404).

Variable	Category	*N*	%
Age	<30	153	37.9%
	30–39	77	19.1%
	40–49	96	23.8%
	50–59	61	15.1%
	>60	17	4.2%
Education	High school	144	35.6%
	Bachelor’s degree	203	50.2%
	Master’s degree	36	8.9%
	PhD	21	5.2%
Salary Income	<5,000	230	56.9%
	5,000–10,000	81	20.0%
	10,001–20,000	63	15.6%
	>20,000	30	7.4%
How do you pay for healthcare?	Government sponsorship	182	45.0%
	Private health insurance	114	28.2%
	Personal payment	108	26.7%
In your opinion, what is the appropriate age to start breast cancer screening for healthy people?	<30	159	39.4%
	30–40	143	35.4%
	40–50	93	23.0%
	>50	9	2.2%
Do you have a first or second-degree relative with breast cancer?	No	287	71.0%
	Yes	117	29.0%
Do you have any history of breast diseases?	No	379	93.8%
	Yes	25	6.2%
Have you discussed breast cancer with a doctor?	No	310	76.7%
	Yes	94	23.3%

Table 1 summarizes knowledge about breast cancer screening. Perceived knowledge was moderate overall (M = 5.26, SD = 2.94). Endorsement of screening benefits was nearly universal across items, whereas practical knowledge showed a gap, with 33.9% reporting that they did not know how to screen for breast cancer.

Table 2 indicates generally favourable attitudes toward breast cancer screening. Most participants supported screening even without symptoms (86.4%) and reported that they would follow a doctor’s recommendation (84.9%). Support for screening after age (45) was also common (65.6%), while scepticism about test effectiveness was uncommon (6.4%), indicating overall high trust in screening.

Table 4 highlights that the most frequently reported barrier was perceiving screening as unnecessary in the absence of symptoms (69.8%). Fear-related barriers were also prominent, particularly fear of examination (55.2%) and fear of the results (48.8%). Other practical and access-related barriers (e.g., time constraints, access, and cost) were reported by substantial proportions.

**Table 4:** Barriers to breast cancer screening (*N* = 404).

Item	No *n* (%)	Yes *n* (%)
What is the main barrier that might prevent you from undergoing breast cancer screening? (You may choose more than one option)		
lack of knowledge	249 (61.6%)	155 (38.4%)
Fear of examination	181 (44.8%)	223 (55.2%)
Cost	246 (60.9%)	158 (39.1%)
Lack of access to screening	235 (58.2%)	169 (41.8%)
Fear of the results	207 (51.2%)	197 (48.8%)
Previous bad experience	333 (82.4%)	71 (17.6%)
Discomfort with doctors	300 (74.3%)	104 (25.7%)
Time constraints	206 (51.0%)	198 (49.0%)
Having no symptoms makes me think I need to get tested.	122 (30.2%)	282 (69.8%)

Participants could select more than one barrier. Each barrier option was coded as Yes if selected and No if not selected.

Table 5 shows that 33.2% of participants reported having had a clinical examination by a specialist, while 21.5% reported regular mammography in the full sample. Because 56.9% of participants were younger than 40 years, this overall mammography percentage should not be interpreted as the uptake rate among women in the routine screening-age group. Nearly two-thirds of participants scored 0 on the screening practices index (64.1%), while 17.1% reported one practice and 18.8% reported both. The mean screening practices score was 0.55 (SD = 0.79; range = 0–2).

**Table 5:** Screening practices (*N* = 404).

Item	Response	n	%
Have you ever had a clinical examination by a specialist?	No	270	66.8%
	Yes	134	33.2%
Have you had a mammogram regularly?	No	317	78.5%
	Yes	87	21.5%
Screening practices score (0–2)	0	259	64.1%
	1	69	17.1%
	2	76	18.8%

The screening practices score was based on two practices: having had a clinical examination by a specialist and having regular mammography. Each practice was coded as 0 = No and 1 = Yes, yielding a total score ranging from 0 to 2.

When regular mammography was examined by age, 43.1% (75/174) of participants aged ≥40 years reported regular mammography, compared with 5.2% (12/230) of those aged <40 years [Pearson’s *χ*^2^(1) = 84.15, *p* < 0.001]. Thus, the 21.5% observed in the full sample primarily reflects the younger age composition of the study sample and should not be interpreted as the mammography uptake rate among participants aged ≥40 years.

Table 6 shows that overall knowledge and pro-screening attitudes were high, with participants scoring near the upper ends of the observed ranges for both the knowledge (M = 6.99, SD = 0.95) and attitude (M = 3.30, SD = 0.88) scores. In contrast, the screening practices score was low (M = 0.55, SD = 0.79), indicating limited engagement in regular screening behaviors despite favorable knowledge and attitudes. Barriers showed moderate endorsement (M = 3.85, SD = 2.29).

**Table 6:** Composite scores (*N* = 404).

Composite score	Possible range	Observed Min	Observed Max	M	SD
Knowledge score	0–8	1	8	6.99	0.95
Pro-screening attitude score	0–4	0	4	3.30	0.88
Barriers score	0–9	0	9	3.85	2.29
Screening practices score	0–2	0	2	0.55	0.79

Values are means (M) and standard deviations (SD).

The barriers score was weakly negatively correlated with knowledge (*ρ* = −0.247, *p* < 0.001), pro-screening attitude (*ρ* = −0.194, *p* < 0.001), and screening practices (*ρ* = −0.106, *p* = 0.032).

Table 7 presents the composite knowledge, pro-screening attitude, and barriers scores according to family history of breast cancer, history of breast disease, and discussion of breast cancer with a doctor. Participants with a history of breast disease had significantly higher knowledge scores than those without such a history (M = 7.52 vs. 6.96, *p* = 0.002). Participants who had discussed breast cancer with a doctor also had significantly higher knowledge scores (M = 7.47 vs. 6.85, *p* < 0.001) and pro-screening attitude scores (M = 3.52 vs. 3.24, *p* = 0.003) than those who had not. No significant differences were found according to family history of breast cancer for any of the three composite scores, and barriers scores did not differ significantly across any of the examined subgroups (all *p* > 0.05).

**Table 7:** Composite knowledge, pro-screening attitude, and barriers scores across selected participant subgroups (*N* = 404).

Subgroup variable	Category	*N*	Knowledge score (0–8), M (SD)	*p*-value	Pro-screening attitude score (0–4), M (SD)	*p*-value	Barriers score (0–9), M (SD)	*p*-value
Family history of breast cancer	No	287	6.94 (0.91)	0.121	3.29 (0.90)	0.490	3.79 (2.28)	0.391
	Yes	117	7.11 (1.03)		3.35 (0.83)		4.01 (2.32)	
History of breast disease	No	379	6.96 (0.95)	0.002	3.30 (0.88)	0.574	3.84 (2.29)	0.760
	Yes	25	7.52 (0.77)		3.40 (0.87)		4.00 (2.45)	
Discussed breast cancer with a doctor	No	310	6.85 (0.97)	<0.001	3.24 (0.91)	0.003	3.95 (2.31)	0.139
	Yes	94	7.47 (0.65)		3.52 (0.76)		3.55 (2.22)	

Values are means (M) and standard deviations (SD) unless otherwise indicated. Unadjusted p-values are from Welch’s independent-samples t-tests comparing the Yes and No categories within each subgroup variable.

Table 8 shows that regular breast cancer screening uptake varied markedly by age and was substantially higher among participants who had discussed breast cancer with a doctor [*χ*^2^(1) = 82.750, *p* < 0.001]. Uptake also differed by income [*χ*^2^(3) = 19.993, *p* < 0.001] and healthcare payment method [*χ*^2^(2) = 6.274, *p* = 0.043], whereas education was not associated with regular breast cancer screening [*χ*^2^(3) = 2.279, *p* = 0.517].

**Table 8:** Bivariate associations with regular breast cancer screening “Have you had a mammogram regularly?” (*N* = 404).

Parameter	Category	No		Yes		*χ*^2^ (df)	*p*-Value
		*N*	%	*N*	%		
Age	<30	147	96.1%	6	3.9%	109.614 (4)	*p* < 0.001
	30–39	71	92.2%	6	7.8%		
	40–49	67	69.8%	29	30.2%		
	50–59	28	45.9%	33	54.1%		
	>60	4	23.5%	13	76.5%		
Education	High school	114	79.2%	30	20.8%	2.279 (3)	*p* = 0.517
	BCs	159	78.3%	44	21.7%		
	MCs	30	83.3%	6	16.7%		
	PhD	14	66.7%	7	33.3%		
Salary Income	<5,000	195	84.8%	35	15.2%	19.993 (3)	*p* < 0.001
	5,000–10,000	51	63.0%	30	37.0%		
	10,001–20,000	45	71.4%	18	28.6%		
	>20,000	26	86.7%	4	13.3%		
How do you pay for healthcare?	Government sponsorship	134	73.6%	48	26.4%	6.274 (2)	*p* = 0.043
	Private health insurance	90	78.9%	24	21.1%		
	Personal payment	93	86.1%	15	13.9%		
Start age opinion	<30	131	82.4%	28	17.6%	8.257 (3)	*p* = 0.041
	30–40	101	70.6%	42	29.4%		
	40–50	78	83.9%	15	16.1%		
	>50	7	77.8%	2	22.2%		
Relatives with breast cancer	No	243	84.7%	44	15.3%	22.571 (1)	*p* < 0.001
	Yes	74	63.2%	43	36.8%		
History of breast diseases	No	303	79.9%	76	20.1%	7.960 (1)	*p* = 0.005
	Yes	14	56.0%	11	44.0%		
Discussion with doctor	No	275	88.7%	35	11.3%	82.750 (1)	*p* < 0.001
	Yes	42	44.7%	52	55.3%		

Values are *n* and (%) within each category. *p*-values are from Pearson’s chi-square test.

Table 9 shows that age was a strong predictor of regular breast cancer screening (overall *p* < 0.001), with markedly lower odds among younger participants compared with those aged >60 (e.g., <30: aOR = 0.013, 95% CI [0.003, 0.065], *p* < 0.001). Discussing breast cancer with a doctor was independently associated with higher odds of regular breast cancer screening (aOR = 5.761, 95% CI [2.732, 12.146], *p* < 0.001). Salary income showed an overall association (*p* = 0.028), and the (5000–10,000) group had higher odds than the (>20,000) group (aOR = 8.709, 95% CI [1.606, 47.211], *p* = 0.012). After adjustment, knowledge, barriers, and perceived appropriate starting age were not associated with regular breast cancer screening (*p* > 0.05), while pro-screening attitude showed a borderline association (*p* = 0.061). Payment method was not associated with regular mammography at the overall factor level (overall *p* = 0.143), despite the nominal category-level contrast for government sponsorship versus personal payment (*p* = 0.049).

**Table 9:** Logistic regression predicting regular breast cancer screening “Have you had a mammogram regularly?” (*N* = 404).

Predictor	Category	aOR	95% CI		Contrast *p*-value	Overall *p*-value
			Lower	Upper		
Age (ref: >60)	<30	0.013	0.003	0.065	<0.001	<0.001
	30–39	0.028	0.006	0.145	<0.001	
	40–49	0.100	0.023	0.427	0.002	
	50–59	0.248	0.056	1.093	0.065	
Education (ref: PhD)	High school	0.821	0.162	4.158	0.811	0.860
	BCs	0.896	0.199	4.036	0.886	
	MCs	0.540	0.091	3.218	0.498	
Salary Income (ref: >20,000)	<5,000	3.798	0.705	20.463	0.120	0.028
	5,000–10,000	8.709	1.606	47.211	0.012	
	10,001–20,000	2.842	0.544	14.845	0.216	
Payment method (ref: Personal payment)	Government sponsorship	2.389	1.004	5.685	0.049	0.143
	Private health insurance	1.858	0.694	4.972	0.217	
Appropriate starting age (ref: >50)	<30	1.263	0.163	9.759	0.823	0.485
	30–40	2.176	0.287	16.491	0.452	
	40–50	1.359	0.171	10.775	0.772	
Family history of breast cancer (ref: No)	Yes	1.566	0.777	3.158	–	0.210
History of breast diseases (ref: No)	Yes	1.251	0.340	4.599	–	0.736
Discussed breast cancer with a doctor (ref: No)	Yes	5.761	2.732	12.146	–	<0.001
Knowledge score (0–8)	Per 1-point increase	1.116	0.740	1.682	–	0.600
Pro-screening attitude score (0–4)	Per 1-point increase	1.481	0.983	2.233	–	0.061
Barriers score (0–9)	Per 1-point increase	1.006	0.864	1.171	–	0.943

confidence interval. Reference categories were age >60 years, PhD education, salary income >20,000 SAR, personal payment, perceived appropriate starting age >50 years, and “No” for binary variables. For multi-category variables, overall *p*-values are based on Wald *χ*^2^ tests, whereas category-level *p*-values represent contrasts with the specified reference category. Overall Wald tests were: age *χ*^2^(4) = 45.257, *p* < 0.001; education *χ*^2^(3) = 0.755, *p* = 0.860; salary income *χ*^2^(3) = 9.081, *p* = 0.028; payment method *χ*^2^(2) = 3.884, *p* = 0.143; and perceived appropriate starting age *χ*^2^(3) = 2.448, *p* = 0.485.

The overall model was statistically significant [Omnibus *χ*^2^(21) = 171.313, *p* < 0.001], with a Nagelkerke R^2^ of 0.534. The Hosmer–Lemeshow test was not statistically significant [*χ*^2^(8) = 14.733, *p* = 0.065], although this result was interpreted cautiously given its proximity to the conventional 0.05 threshold. The model showed good discrimination (AUC = 0.888), and VIF values ranging from 1.05 to 1.32 indicated no evidence of problematic multicollinearity. Sensitivity analyses using alternative cut-offs (≥7) for the two dichotomized 10-point items yielded materially similar estimates and did not change the substantive interpretation of the main findings (Table 10).

**Table 10:** Sensitivity analysis comparing the primary and alternative cut-off specifications.

Model result	Primary specification	Alternative cut-offs (≥7)
Age, overall *p*-value	<0.001	<0.001
Salary income, overall *p*-value	0.028	0.029
Discussion with doctor, aOR (95% CI), *p*-value	5.761 (2.732–12.146), <0.001	5.621 (2.695–11.724), <0.001
Knowledge score, aOR (95% CI), *p*-value	1.116 (0.740–1.682), 0.600	1.215 (0.801–1.842), 0.360
Pro-screening attitude score, aOR (95% CI), *p*-value	1.481 (0.983–2.233), 0.061	1.393 (0.919–2.111), 0.118
Barriers score, aOR (95% CI), p-value	1.006 (0.864–1.171), 0.943	1.012 (0.868–1.179), 0.881
Nagelkerke *R*^2^	0.534	0.533
AUC	0.888	0.888

Both models included the same covariates. The primary specification used the prespecified cut-offs reported in Tables 1, 2, whereas the sensitivity specification used a cut-off of ≥7 for both 10-point items. The substantive interpretation of the main predictors was unchanged.

## 4 Discussion

Breast cancer screening is essential to cancer control, especially in nations where it is the primary cause of morbidity among females (21). Knowledge gaps, cultural views, and healthcare system constraints affect Saudi adult breast cancer screening adherence. National awareness efforts and expanded healthcare services have raised breast cancer awareness, but screening program participation remains low. Breast cancer screening knowledge and favorable attitudes may not be enough to assure adherence since psychological, social, cultural, and practical constraints influence women’s screening behavior (16). In a previous study, Abdul Rehman et al. (22), A total of 37 studies were eligible. They found eight hurdles to BCa-related care for Pakistani women: lack of awareness, accessibility, affordability, fear, reluctance, cultural customs, commitments, and medical mistakes. Most commonly mentioned factors were lack of understanding, cultural norms, fear, and inadequate accessibility, with physician error contributing the least. Barriers, such as poverty preventing access to healthcare, create complex and multifaceted situations. In addition, another study Ponce-Chazarri et al. (23), most have common barriers like race/ethnicity, low socioeconomic and educational levels, no family history of cancer and being single, medical mistrust and a health information gap, lack of private health insurance and not having annual health checks.

This study aims to evaluate compliance with breast cancer screening standards among adult women in the Central Region (Riyadh) of Saudi Arabia and to identify the primary obstacles that impede successful participation in these screening programs.

The demographic profile suggests that the sample was largely composed of younger and middle-aged adults, with relatively fewer older participants. Educational attainment was generally moderate to high, with many participants holding undergraduate qualifications, while postgraduate education was less prevalent. Despite this educational background, household income was predominantly low, and reliance on government-sponsored healthcare was common, reflecting the broader context of healthcare access and utilization within the population.

Education and occupational level may influence participation in breast cancer screening programs by shaping women’s health awareness, access to information, and ability to seek preventive care. Women with higher educational attainment are often more familiar with the benefits of early detection, while occupational status may affect both access to health services and available time to attend screening appointments. These factors can therefore contribute to differences in awareness, attitudes, and adherence to breast cancer screening practices (24).

Given that the sample included a relatively high proportion of women with undergraduate education, estimates of knowledge and positive perceptions of screening benefits may be higher than would be observed in samples with lower educational attainment, and should therefore be generalized cautiously. In addition, because many participants were younger than the age group typically targeted for routine mammography, the overall prevalence of regular mammography may underestimate uptake among women aged ≥40 years. As shown in the Results, regular mammography was substantially more common among participants aged ≥40 years than among those aged <40 years (43.1% vs. 5.2%). Accordingly, the overall prevalence of 21.5% should not be interpreted as the mammography uptake rate among the older screening-age subgroup. The national Saudi breast cancer screening guidelines include appropriate age groups, screening techniques, and how early diagnosis reduces disease burden. Participants demonstrated generally favorable perceptions toward breast cancer screening. Attitudes were strongly supportive, with widespread agreement that screening is important even in the absence of symptoms and that medical recommendations should be followed. Trust in the effectiveness of screening was also high, indicating that skepticism toward screening as a concept is not a major obstacle. This positive attitudinal profile is consistent with the high endorsement of screening benefits, suggesting that public health messaging about the value of early detection has been largely effective. The percentages among participants were quite similar, indicating that an individual’s view of the usefulness and efficacy of cancer screening tests influences their acceptance, hence corroborating our findings (25, 26). However, despite high perceived benefits and supportive attitudes, actual screening practices were limited. Most participants reported no engagement in either breast cancer screening or regular mammography. This discrepancy highlights a well-recognized knowledge–practice gap, in which positive beliefs do not necessarily translate into preventive action. Although perceived knowledge was moderate overall, substantial variability was observed, with many participants rating their knowledge as low. Moreover, while awareness of screening benefits was nearly universal, practical knowledge, such as knowing how to perform screening, was notably weaker. This distinction underscores the importance of differentiating between abstract awareness and actionable knowledge (27, 28).

Perceived barriers were commonly reported and may help characterize the context in which screening decisions occur, but their role should be interpreted cautiously. The most frequently endorsed barrier was the belief that screening is unnecessary in the absence of symptoms, while fear of examination, fear of results, time constraints, access, cost, and insufficient knowledge were also frequently reported. At the composite-score level, higher barriers scores were only weakly associated with lower knowledge, pro-screening attitudes, and screening practices, and the barriers score was not independently associated with regular mammography after multivariable adjustment. These findings therefore support describing barriers as commonly reported concerns rather than as factors that independently explain the knowledge–practice gap.

The analysis of factors associated with regular breast cancer screening revealed that age was a particularly strong determinant, with uptake increasing markedly among older participants. This pattern likely reflects greater perceived vulnerability, increased exposure to healthcare services, and stronger alignment with age-based screening recommendations. Discussion of breast cancer with a physician emerged as the most influential modifiable factor, strongly associated with higher screening uptake. However, because physician discussion and screening were assessed cross-sectionally, their temporal ordering cannot be established; physician discussion may have preceded screening, but it may also have occurred after screening or in response to breast symptoms or concerns. Therefore, this association should be interpreted as a correlate of screening uptake rather than evidence that physician discussion caused screening participation. Those over 45 should get a mammogram every year or two, while those under 45 should get one every year (29).

In contrast, education level was not associated with regular breast cancer screening, indicating that formal education alone may be insufficient to drive screening behavior. Similarly, after adjustment, knowledge scores and perceived barriers were not independently associated with regular breast cancer screening, indicating that their observed relationships with screening-related behaviors were not retained after accounting for the other variables in the model. In addition, some adjusted estimates may have wide confidence intervals due to sparse data in certain categories and should therefore be interpreted cautiously. Pro-screening attitude showed a borderline but statistically non-significant adjusted association with regular breast cancer screening (*p* = 0.061). Breast cancer screening utilization rates were similarly low in other research in Africa (30, 31) and Asia (32).

## 5 Limitation

This study has several limitations that should be considered when interpreting the findings. First, the cross-sectional design limits the ability to establish causal relationships between knowledge, attitudes, perceived barriers, and breast cancer screening practices. The associations identified reflect correlations at a single point and cannot determine whether these factors directly influenced screening behavior. This limitation is particularly relevant to the association between physician discussion and screening: women may have discussed breast cancer with a physician before screening, but such discussions may also have occurred after screening or in response to breast symptoms or concerns, making reverse causation possible. Second, the use of self-reported data may have introduced recall and social desirability biases, as participants may have overestimated positive attitudes or underreported barriers and nonadherence to screening recommendations. In addition, the barriers index was based on a summed count of heterogeneous barrier options and therefore may not fully capture the severity or dimensional structure of perceived barriers. Third, A limitation of this study is the potential sampling bias resulting from the online recruitment strategy, which relied on Google Forms, social media, and snowball sampling. This method may have preferentially included younger, more educated, and more digitally literate women, thereby affecting the estimated adherence rates, particularly the low observed screening uptake. In addition, because the sample was skewed toward younger participants, many respondents may not have been in the age group typically eligible for routine breast cancer screening, potentially leading to an underestimation of adherence. Another limitation is that the study did not explore in depth the role of healthcare system factors, such as the availability of female healthcare providers, access to appointments, or the quality of physician counselling. The quality of physician counseling may be an important factor associated with screening participation, as effective counseling can shape patients’ understanding, perceptions, and willingness to engage in screening.

Another critical limitation of this study is the potential presence of selection bias and the limited generalizability of the findings. Participants were recruited primarily through university mailing lists, faculty WhatsApp groups, and social media platforms, which may have resulted in an overrepresentation of younger, highly educated women. Notably, a substantial proportion of the sample was under 30 years of age, whereas the national breast cancer screening program in Saudi Arabia primarily targets women aged 40–69 years. Because regular breast cancer screening uptake was a main outcome of interest, the underrepresentation of women in the recommended screening-age group limits the extent to which the findings can be generalised to real-world screening adherence among the target population.

Additionally, another limitation is that the response rate was not calculated because the survey was distributed through open online channels, including university networks, WhatsApp groups, and social media platforms, where the total number of individuals who received or viewed the survey link could not be accurately determined. This recruitment approach may have introduced recruitment-related bias, as participation was more likely among individuals with internet access, higher digital literacy, links to academic or university communities, and a greater interest in breast cancer awareness or screening. Consequently, the sample may not fully represent the broader Saudi female population, particularly women outside university-affiliated networks or those with limited access to online platforms.

Despite these limitations, the study provides valuable insight into important barriers and factors affecting breast cancer screening adherence in this population. These findings may guide national screening policy by identifying population groups with lower breast cancer screening uptake and highlighting the need for targeted awareness campaigns, improved access, and stronger physician–patient communication within organized screening programs.

## 6 Conclusion

These findings highlight the need for interventions that move beyond raising awareness and instead focus on translating positive attitudes into action. Strategies should strengthen physician–patient communication and address commonly reported symptom-based misconceptions and emotional or practical concerns related to screening. Tailored communication, reassurance, and system-level facilitation may be particularly effective in bridging the gap between knowledge and practice and improving breast cancer screening uptake.

## Data Availability

The datasets presented in this study can be found in online repositories. The names of the repository/repositories and accession number(s) can be found in the article/Supplementary material.
