# Supplementary material for: Assessing adherence and identifying barriers to breast cancer screening among females in the central region (Riyadh) of Saudi Arabia: a cross-sectional study
Source: Front Public Health. 2026 Oct 15;14:1924881. doi: 10.3389/fpubh.2026.1924881 (PMC13716766; doi:10.3389/fpubh.2026.1924881)
Supplement: Supplementary file 1 [file Data_Sheet_1.PDF]

Assessing Adherence and Identifying Barriers to Breast Cancer Screening in the Adult General Populations of Saudi تقييم الالتزام بفحص سرطان الثدي وتحديد عوائقه لدى عموم السكان البالغين في المملكة العربية السعودية: دراسة مقطعية

استبيان: تقييم الالتزام وتحديد العوائق أمام فحص سرطان الثدي

شكرًا لمشاركتك في هذه الدراسة البحثية التي تهدف
إلى تقييم الالتزام بفحص سرطان الثدي وتحديد العوائق التي قد تحول دون
إجرائه بين عامة السكان في المملكة العربية السعودية. نؤكد أن جميع المعلومات ستُستخدم
.لأغراض البحث فقط، وستُحفظ بسرية تامة

سرطان الثدي هو أحد أكثر أنواع السرطان شيوعًا، ويمكن الوقاية منه بالكشف المبكر من خلال
فحوصات مبكرةمثل الفحص الذاتي الشهري، والفحص السريري لدى طبيبة
متخصصة، وتصوير الثدي بالماموجرام بشكل دوري لقد قرأت
:المعلومات الموضحة أعلاه، وأقر بالمشاركة في هذا المشروع البحثي

Statement: Assessing Adherence and Identifying Barriers to Breast Cancer Screening

Thank you for participating in this research study, which aims to assess adherence to breast cancer screening and identify barriers to its implementation among the general population in the Kingdom of Saudi Arabia. We assure you that all information will be used for research purposes .only and will be kept strictly confidential

Breast cancer is one of the most common types of cancer and can be prevented through early detection through screening tests such as monthly self-examination, clinical examination by a specialist, and regular mammograms. I have read the information above

* تشير إلى أنّ السؤال مطلوب

1. الجنس *

Gender

حدد دائرة واحدة فقط.

- Female أنثى
- ذكر Male

2. agree to participate in this research project*
هل توافق بالمشاركة في اكمال الاستبيان البحثي

حدد دائرة واحدة فقط.

- Yes نعم
- No لا

3. العمر *

Age

حدد دائرة واحدة فقط.

- < 30
- 30-39
- 40-49
- 50-59
- >60

4. المستوى التعليمي*

Education

حدد دائرة واحدة فقط.

- ◯ خريج ثانوية عامة High school
- ◯ شهادة البكالوريوس BCs
- ◯ شهادة الماجستير MCs
- ◯ شهادة الدكتوراه PhD

5. مستوى الدخل الشهري*

Salary Income

حدد دائرة واحدة فقط.

- ◯ < 5000
- ◯ 5000 - 10000
- ◯ 10001 - 20000
- ◯ > 20000

6. كيف تدفع تكاليف الرعاية الصحية؟ *
How do you pay for healthcare?

حدد دائرة واحدة فقط.

- ◯ تأمين صحي خاص private health insurance
- ◯ رعاية حكومية Government sponsorship
- ◯ دفع شخصي Personal payment

7. هل لديك قريب من الدرجة الأولى أو الثانية مصاب بسرطان الثدي؟ *
Do you have a first or second-degree relative with breast cancer?

حدد دائرة واحدة فقط.

- نعم Yes
- No لا

8. هل لديك أي تاريخ مرضي بأمراض الثدي ؟ *
Do you have any history of breast diseases?

حدد دائرة واحدة فقط.

- نعم Yes
- No لا

9. هل ناقشت سرطان الثدي مع طبيب؟ *

Have you discussed breast cancer with a doctor?

حدد دائرة واحدة فقط.

- نعم Yes
- No لا

10. على مقياس من 1 (لا أعرف شيئًا) إلى 10 (أعرف الكثير) *

كيف تقيم معرفتك بفحص سرطان الثدي؟
On a scale of 1 (I don't know anything) to 10 (I know a lot),
how would you rate your knowledge of breast cancer screening

حدد دائرة واحدة فقط.

1	2	3	4	5	6	7	8	9	10
○	○	○	○	○	○	○	○	○	○

11. ما فائدة فحص سرطان الثدي؟ *
What is the benefit of breast cancer screening?

حدد دائرة واحدة فقط في كل صف.

	Yes نعم	No لا	
اكتشاف سرطان الثدي في مرحلة مبكرة Early detection of breast cancer	⬭	⬭	اكتشاف سرطان الثدي في مرحلة مبكرة Early detection of breast cancer
زيادة فرص الشفاء والعلاج الناجح Improving chances of cure and successful treatment	⬭	⬭	زيادة فرص الشفاء والعلاج الناجح Improving chances of cure and successful treatment
تقليل مخاطر الوفاة بسبب سرطان الثدي Reducing the risk of death from breast cancer	⬭	⬭	تقليل مخاطر الوفاة بسبب سرطان الثدي Reducing the risk of death from breast cancer
الاطمئنان عند ظهور نتيجة طبيعية للفحص Providing reassurance when results are normal	⬭	⬭	الاطمئنان عند ظهور نتيجة طبيعية للفحص Providing reassurance when results are normal
اكتشاف تغييرات أو أورام قد تصبح خطيرة لاحقًا Detecting changes or lumps that may become dangerous later	⬭	⬭	اكتشاف تغييرات أو أورام قد تصبح خطيرة لاحقًا Detecting changes or lumps that may become dangerous later
منع تطور السرطان	⬭	⬭	منع تطور السرطان

Preventing the development of cancer	Preventing the development of cancer

12. في رأيك، ما هو العمر المناسب لبدء فحص سرطان الثدي للأشخاص الأصحاء *

In your opinion, what is the appropriate age to start breast cancer screening for healthy people?

حدد دائرة واحدة فقط.

- <30
- 30-40
- 40- 50
- >50

13. هل تعرف طرق فحص سرطان الثدي؟ *

Do you know how to screen for breast cancer?

حدد دائرة واحدة فقط.

- نعم yes
- No لا

14. هل تعتقد أن فحص سرطان الثدي يجب أن يُجرى حتى لو لم يكن لديك أعراض؟ *

Do you think breast cancer screening should be done even if you don't have symptoms?

حدد دائرة واحدة فقط.

- نعم yes
- No لا

15. على مقياس من 1 (لا أعرف شيئًا) إلى 10 (أعرف الكثير) *

On a scale of 1 (I don't know anything) to 10 (I know a lot),
إلى أي مدى تؤيد إجراء الفحص بعد سن 45؟
How strongly do you support screening after age 45?

حدد دائرة واحدة فقط.

1	2	3	4	5	6	7	8	9	10
◯	◯	◯	◯	◯	◯	◯	◯	◯	◯

16. إذا أوصى طبيبك بإجراء الفحص، هل ستلتزم به؟ *

If your doctor recommends a test, will you follow it?

حدد دائرة واحدة فقط.

- ◯ نعم yes
- ◯ Not sure غير متأكد
- ◯ لا No

17. هل تشكك في فعالية الفحص؟ *

Do you question the effectiveness of the test?

حدد دائرة واحدة فقط.

- ◯ نعم yes
- ◯ Not sure غير متأكد
- ◯ لا No

18. ما هو العائق الرئيسي الذي قد يمنعك من إجراء فحص سرطان الثدي (يمكنك اختيار أكثر من خيار) *

What is the main barrier that might prevent you from undergoing breast cancer screening? (You may choose more than one option)

حدد دائرة واحدة فقط في كل صف.

	yes نعم	No لا	
نقص المعرفة lack of knowledge			نقص المعرفة lack of knowledge
الخوف من الفحص Fear of examination			الخوف من الفحص Fear of examination
التكلفة Cost			التكلفة Cost
عدم وجود إمكانية الوصول للفحص Lack of access to screening			عدم وجود إمكانية الوصول للفحص Lack of access to screening
الخوف من النتيجة Fear of the results			الخوف من النتيجة Fear of the results
تجربة سيئة سابقة previous bad experience			تجربة سيئة سابقة previous bad experience
عدم الراحة مع الأطباء discomfort with doctors			عدم الراحة مع الأطباء discomfort with doctors
ضيق الوقت time constraints			ضيق الوقت time constraints
عدم وجود أعراض تجعلني لا أعتقد أنني بحاجة للفحص Having no symptoms			عدم وجود أعراض تجعلني لا أعتقد أنني بحاجة للفحص Having no symptoms makes me

makes me not think I need to get tested.	not think I need to get tested.

19. هل أجريت الفحص السريري لدى طبيبة متخصصة من قبل؟ *

Have you ever had a clinical examination by a specialist?

حدد دائرة واحدة فقط.

- نعم Yes
- No لا

20. هل أجريت تصوير الثدي بالماموجرام بشكل دوري ؟ *

Have you had a mammogram regularly?

حدد دائرة واحدة فقط.

- نعم yes
- No لا

21. Do you think, Mammograms should be done every two years for women 40-50.
هل تعتقد أنه يجب إجراء تصوير الثدي بالأشعة بالماموجرام كل عامين للنساء في سن 40-50؟

حدد دائرة واحدة فقط.

- Yes نعم
- No لا

لم يتم إنشاء هذا المحتوى ولا اعتماده من قِبل Google.

Google نماذج
